# Maximum likelihood estimation of log-affine models using detailed-balanced reaction networks

**DOI:** 10.1007/s00285-025-02262-5

**Published:** 2025-09-10

**Authors:** Oskar Henriksson, Carlos Améndola, Jose Israel Rodriguez, Polly Y. Yu

**Affiliations:** 1 Department of Mathematical Sciences, University of Copenhagen, Copenhagen, Denmark; 2 Institute of Mathematics, Technical University of Berlin, Berlin, Germany; 3 Department of Mathematics and Department of Electrical and Computer Engineering, University of Wisconsin – Madison, Madison, USA; 4 Department of Mathematics, University of Illinois Urbana-Champaign, Urbana, USA

**Keywords:** 68Q07, 62F10, 62R01, 14M25, 92C42, 92E20

## Abstract

A fundamental question in the field of molecular computation is what computational tasks a biochemical system can carry out. In this work, we focus on the problem of finding the maximum likelihood estimate (MLE) for log-affine models. We revisit a construction due to Gopalkrishnan of a mass-action system with the MLE as its unique positive steady state, which is based on choosing a basis for the kernel of the design matrix of the model. We extend this construction to allow for any finite spanning set of the kernel, and explore how the choice of spanning set influences the dynamics of the resulting network, including the existence of boundary steady states, the deficiency of the network, and the rate of convergence. In particular, we prove that using a Markov basis as the spanning set guarantees global stability of the MLE steady state.

## Introduction

A recent line of research in synthetic and systems biology is *molecular computation*, which studies the problem of designing biological systems for carrying out given computations, including arithmetic (Anderson and Joshi 2024), evaluation of algebraic functions (Buisman et al. 2009) or piecewise functions like $\min$ and $\max$ (Chen et al. 2023), approximating statistical distributions (Cappelletti et al. 2020), parameter inference (Gopalkrishnan 2016; Virinchi et al. 2017, 2018; Wiuf et al. 2023), and even implementing simple neural networks (Anderson et al. 2021). A common approach is to construct a chemical reaction network obeying mass-action kinetics, for which the steady state is the solution to the computational problem. Realizing such reaction networks *in vivo* is becoming more feasible with emerging technology, for example by DNA strand replacement techniques (Soloveichik et al. 2010; Qian et al. 2010; Chen et al. 2013; Srinivas 2015; Lv et al. 2021).

In this work, we focus on the statistical problem of maximum likelihood estimation, for models that are *log-affine*. This includes many important models, for instance graphical models (see Lauritzen 1996) and hierarchical models in contingency tables (Hara et al. 2012). A log-affine model with *m* states and *d* parameters is given by a design matrix $\textbf{A}=({\varvec{a}}_1,\ldots ,{\varvec{a}}_m)\in \mathbb {Z}^{d\times m}$ and a scaling vector ${\varvec{c}}\in \mathbb {R}_{>0}^m$, such that the set of possible probability distributions takes the form

$$\begin{aligned} \mathcal {M}_{\textbf{A},{\varvec{c}}} = \left\{ (c_1 {\varvec{t}}^{{\varvec{a}}_1}, \dots , c_m {\varvec{t}}^{{\varvec{a}}_m}): {\varvec{t}} \in \mathbb {R}_{>0}^d, \, c_1 {\varvec{t}}^{{\varvec{a}}_1}+ \dots + c_m {\varvec{t}}^{{\varvec{a}}_m}=1 \right\} , \end{aligned}$$

where we use the notation ${\varvec{t}}^{{\varvec{b}}} = t_1^{b_1}\cdots t_d^{b_d}$ for vectors ${\varvec{t}}=(t_1,\ldots ,t_d)$ and ${\varvec{b}}=(b_1,\ldots ,b_d)$.

The problem of maximum likelihood estimation by a reaction network was first approached by Gopalkrishnan (2016). Specifically, it gives a mass-action system whose unique positive steady state is the maximum likelihood estimate (MLE), when the initial concentrations are given by the normalized observed distribution. A crucial part of this construction is a choice of $\mathbb {Z}$-basis $\Lambda$ for the right integer kernel $\ker _{\mathbb {Z}}(\textbf{A})$. In this paper, we extend the construction to allow for any finite (and possibly linearly dependent) set $\Lambda \subseteq \ker _{\mathbb {Z}}(\textbf{A})$ that spans $\ker _{\mathbb {R}}(\textbf{A})$ as a vector space, and show how such $\Lambda$ can give rise to more well-behaved dynamics.

One of the main questions we address concerns the global stability of the MLE steady state. We prove that the mass-action systems of our construction are always *detailed-balanced*. Such systems are known to be asymptotically stable, and we show in Theorem 5.2 that the MLE is guaranteed to be a global attractor if the spanning set $\Lambda$ in the construction is sufficiently large; more precisely, if $\Lambda$ is a *Markov basis*, which is a key concept in algebraic statistics (see, e.g., Almendra-Hernández et al. 2024 for a review). The key step in the proof is to rule out relevant boundary steady states, and the importance of the Markov basis assumption for this is highlighted in Example 5.4. With this, we correct an inaccuracy in Lemma 2 and Theorem 5 of Gopalkrishnan (2016), which claimed that choosing an integer basis for $\ker _{\mathbb {Z}}(\textbf{A})$ suffices for ruling out relevant boundary steady states.

A key geometric feature of log-affine models that is used in the construction and our global stability proof is that $\mathcal {M}_{\textbf{A},{\varvec{c}}}$ can be seen as the intersection of a toric variety with the probability simplex. Toric varieties are central objects in applied algebraic geometry and have a rich combinatorial structure (see, e.g., (Michałek and Sturmfels 2021, Sect. 8.3) for an introduction). A fundamental property of toric varieties that we are using, is that they can be realized as zero sets of finite collections of binomials, and this is precisely what Markov bases encode.

The use of toric varieties in algebraic statistics has a long history; for instance, they have been used for sampling (Diaconis and Sturmfels 1998), decomposability of graphical models (Geiger et al. 2006), and maximum-likelihood estimation (Fienberg and Rinaldo 2012; Améndola et al. 2019, 2020; Davies et al. 2023); see also (Drton et al. 2008) for an overview of other applications. On the reaction networks side, the use of toric geometry dates back to a series of works by Gatermann and coauthors (Gatermann and Huber 2002; Gatermann et al. 2005; Gatermann and Wolfrum 2005), and has more recently centered around the concept of toric dynamical systems (Craciun et al. 2009; Moncusí et al. 2022; Craciun et al. 2020, 2025) (see also the overview in Feliu and Shiu (Feliu and Shiu 2025, Sect. 2)), conditions for monomial parametrizability (see, e.g., Millán 2012; Müller and Regensburger 2012; Millán and Dickenstein 2018; Conradi et al. 2019; Feliu and Henriksson 2024), and reconstruction of networks from single-cell data (Wang et al. 2019). The many toric analogies between reaction network theory and algebraic statistics have previously been discussed in Craciun et al. (2009), and will also be highlighted in the present paper.

Our paper is structured as follows. We recall some of the key relevant concepts from algebraic statistics and reaction network theory in Sects. 2 and 3 respectively, in order to fix notation and make the paper accessible to a broader audience. In Sect. 4, we present our construction of a reaction network based on a spanning set for the kernel of the design matrix, and prove that its unique positive steady state is the MLE of the corresponding log-affine model and data. Section 5 explores the impact the choice of spanning set has on the properties of the system, including global stability, rate of convergence, and the deficiency of the reaction network. We end with a discussion and some outlooks in Sect. 6.

### Notation

In what follows, $\mathbb {R}_{\ge 0}$ denotes the set of nonnegative reals, $\mathbb {R}_{>0}$ the set of positive real numbers, and similarly $\mathbb {Z}_{\ge 0}$, $\mathbb {Z}_{>0}$ denote the sets of nonnegative and positive integers, respectively. We also extend the above notations to vectors, and let $\mathbb {R}_{\ge 0}^m$ denote the nonnegative orthant and $\mathbb {R}_{>0}^m$ the positive orthant. We write $\partial \mathbb {R}_{\ge 0}^m=\mathbb {R}_{\ge 0}^m\setminus \mathbb {R}_{>0}^m$ for the boundary of the nonnegative orthant. For $n\in \mathbb {Z}_{>0}$, we use the notation $[n]=\{1,\ldots ,n\}$. For a vector ${\varvec{x}}\in \mathbb {R}^m$, we write ${\varvec{x}}^+$ for its nonnegative part, and ${\varvec{x}}^-$ for the nonpositive part; in particular, ${\varvec{x}}= {\varvec{x}}^+-{\varvec{x}}^-$. For a matrix $\textbf{A}$, we write $\ker _\mathbb {Z}(\textbf{A})$ to denote the right kernel over the integers, and $\ker (\textbf{A})$ to denote the right kernel over the real numbers. We will also make extensive use of several componentwise operations:

- multiplication: $${\varvec{x}}\circ {\varvec{y}}= (x_1y_1,x_2y_2, \ldots , x_my_m)$$ where $${\varvec{x}}\in \mathbb {R}^m$$, $${\varvec{y}}\in \mathbb {R}^m$$,
- exponentiation: ${\varvec{x}}^{{\varvec{y}}} = x_1^{y_1}x_2^{y_2}\cdots x_m^{y_m}$ where ${\varvec{x}}\in \mathbb {R}_{\ge 0}^m$, ${\varvec{y}}\in \mathbb {R}^m$, and by convention $0^0 = 1$,
- $${{\varvec{t}}}^{\textbf{A}} = ({{\varvec{t}}}^{{\varvec{a}}_1}, {{\varvec{t}}}^{{\varvec{a}}_2}, \ldots , {{\varvec{t}}}^{{\varvec{a}}_m})$$, where $${{\varvec{t}}} \in \mathbb {R}_{\ge 0}^d$$ and $$\textbf{A} = \begin{pmatrix} {\varvec{a}}_1,{\varvec{a}}_2,\ldots , {\varvec{a}}_m\end{pmatrix} \in \mathbb {R}^{d \times m}$$,
- $$\exp ({\varvec{x}}) = (e^{x_1},e^{x_2},\ldots , e^{x_m})$$ where $${\varvec{x}}\in \mathbb {R}^m$$,
- $$\log ({\varvec{x}}) = (\log x_1,\log x_2,\ldots , \log x_m)$$ where $${\varvec{x}}\in \mathbb {R}_{>0}^m$$.

## Algebraic statistics: log-affine models and Birch’s Theorem

Algebraic statistics uses tools from combinatorics, commutative algebra, invariant theory, and algebraic geometry to study problems arising from probability and statistics. In this section we review important concepts from algebraic statistics like log-affine models, Markov bases, and Birch’s Theorem. For a textbook reference, see (Sullivant 2018, Chapters 6–7).

A probability distribution on a finite state space of size $m \in \mathbb {Z}_{>0}$ is determined by probabilities $p_j \ge 0$ for each $j=1,\dots ,m$, and can hence be seen as an element of the $(m-1)$-dimensional probability simplex

$$\begin{aligned} \Delta _{m-1} {:}{=}\left\{ (p_1,p_2,\ldots , p_m) \in \mathbb {R}_{\ge 0}^m : \sum _{i=1}^m p_i = 1 \right\} . \end{aligned}$$

A subset of the probability simplex is called an algebraic statistical model when it is parameterized by a polynomial map or when an implicit description is given by polynomials. The focus of this paper is a particularly well-studied class of algebraic statistical models known as *log-affine models*; see, e.g., (Haber 1985; Stephen 2007; Fienberg and Rinaldo 2012), and (Sullivant 2018, Sect. 6.2).

### Definition 2.1

Given a design matrix $$\textbf{A} = \begin{pmatrix} {\varvec{a}}_1, {\varvec{a}}_2,\ldots , {\varvec{a}}_m\end{pmatrix} \in \mathbb {Z}^{d \times m}$$ that is assumed to contain the vector $\mathbbm {1} {:}{=}(1, 1, \dots , 1)$ in its rowspan, and ${\varvec{c}} \in \mathbb {R}_{>0}^m$, the associated log-affine model is

$$\begin{aligned} \mathcal {M}_{\textbf{A},{\varvec{c}}} {:}{=}\{ {\varvec{p}} \in {{\,\textrm{int}\,}}(\Delta _{m-1}) : \log {\varvec{p}} \in \log {\varvec{c}} + {{\,\textrm{row}\,}}({\textbf{A}}) \} . \end{aligned}$$

In the case when ${\varvec{c}} = \mathbbm {1}$, the model $\mathcal {M}_{\textbf{A}, \mathbbm {1}}$, denoted $\mathcal {M}_{\textbf{A}}$, is said to be log-linear.

To see that a log-affine model is an algebraic statistical model, consider the following description of $\mathcal {M}_{\textbf{A},{\varvec{c}}}$ via the monomial map

2.1 $$\begin{aligned} \varphi _{\textbf{A},{\varvec{c}}}:\mathbb {R}_{>0}^d\rightarrow \mathbb {R}_{>0}^m,\quad {\varvec{t}}\mapsto {\varvec{c}}\circ {\varvec{t}}^{\textbf{A}}=(c_1 {\varvec{t}}^{{\varvec{a}}_1}, \dots , c_m {\varvec{t}}^{{\varvec{a}}_m})\,. \end{aligned}$$

Its image is a scaled positive toric variety, which we denote as $\mathcal {X}_{\textbf{A},{\varvec{c}}}$ (or $\mathcal {X}_{\textbf{A}}$ when ${\varvec{c}}=\mathbbm {1}$), i.e.,

2.2 $$\begin{aligned} \mathcal {X}_{\textbf{A},{\varvec{c}}}=\left\{ (c_1 {\varvec{t}}^{{\varvec{a}}_1}, \dots , c_m {\varvec{t}}^{{\varvec{a}}_m}) : {\varvec{t}} \in \mathbb {R}_{>0}^d \right\} \subseteq \mathbb {R}_{>0}^m. \end{aligned}$$

The model $\mathcal {M}_{\textbf{A},{\varvec{c}}}$ is obtained by restricting $\mathcal {X}_{\textbf{A},{\varvec{c}}}$ to the probability simplex:

$$\begin{aligned} \mathcal {M}_{\textbf{A},{\varvec{c}}} = \mathcal {X}_{\textbf{A},{\varvec{c}}}\cap \Delta _{m-1}= \left\{ (c_1 {\varvec{t}}^{{\varvec{a}}_1}, \dots , c_m {\varvec{t}}^{{\varvec{a}}_m}): {\varvec{t}} \in \mathbb {R}_{>0}^d, \, c_1 {\varvec{t}}^{{\varvec{a}}_1}+ \dots + c_m {\varvec{t}}^{{\varvec{a}}_m}=1 \right\} . \end{aligned}$$

After logarithmizing, $\mathcal {X}_{\textbf{A},{\varvec{c}}}$ is an affine set. From this, we easily obtain an implicit description in terms of binomials; for any set $\Lambda \subseteq \ker _{\mathbb {Z}}(\textbf{A})$ that spans $\ker (\textbf{A})$ as a vector space, it holds that

2.3 $$\begin{aligned} \mathcal {X}_{\textbf{A},{\varvec{c}}}= \left\{ {\varvec{x}}\in \mathbb {R}_{>0}^m : {\varvec{c}}^{{\varvec{\gamma }}^+}{\varvec{x}}^{{\varvec{\gamma }}^-}={\varvec{c}}^{{\varvec{\gamma }}^-}{\varvec{x}}^{{\varvec{\gamma }}^+}\text {for all}\,\, {\varvec{\gamma }}\in \Lambda \right\} . \end{aligned}$$

Since we want to treat distributions where some entries are zero, we also consider the Euclidean closure of $\mathcal {X}_{\textbf{A},{\varvec{c}}}$ in $\mathbb {R}_{\ge 0}^m$:

$$\begin{aligned} \overline{\mathcal {X}}_{\textbf{A},{\varvec{c}}}{:}{=}\overline{\left\{ (c_1 {\varvec{t}}^{{\varvec{a}}_1}, \dots , c_m {\varvec{t}}^{{\varvec{a}}_m}): {\varvec{t}} \in \mathbb {R}_{>0}^d \right\} }\subseteq \mathbb {R}_{\ge 0}^m. \end{aligned}$$

Obtaining an implicit description of $\overline{\mathcal {X}}_{\textbf{A},{\varvec{c}}}$ is a more subtle task than for $\mathcal {X}_{\textbf{A},{\varvec{c}}}$, since we cannot logarithmize vectors with zeroes. In particular, the binomials given by a spanning set $\Lambda$ as in (2.3) might cut out too large a subset of $\mathbb {R}_{\ge 0}^m$ (see Example 2.4 below). Nevertheless, the Zariski closure of $\mathcal {X}_{\textbf{A},{\varvec{c}}}$ in $\mathbb {C}^m$ is a scaled toric variety, so it follows from the theory of toric ideals that there is an implicit description in terms of binomials, given by what is called a Markov basis.

### Definition 2.2

A Markov basis for a design matrix $\textbf{A}\in \mathbb {Z}^{d\times m}$ is a finite set $\Lambda \subseteq \ker _{\mathbb {Z}}(\textbf{A})$ such that the binomials ${\varvec{x}}^{{\varvec{\gamma }}^+}-{\varvec{x}}^{{\varvec{\gamma }}^-}$ for ${\varvec{\gamma }}\in \Lambda$ generate the ideal $I(\mathcal {X}_{\textbf{A}})\subseteq \mathbb {R}[x_1,\ldots ,x_m]$ of all polynomials that vanish on $\mathcal {X}_{\textbf{A}}$.

That such a finite generating set exists for any design matrix follows from Hilbert’s Basis Theorem and (Cox et al. 2011, Proposition 1.1.9). The concept of Markov bases has a long history in algebraic statistics (Almendra-Hernández et al. 2024), and there are many software packages available for computing a Markov basis for a given design matrix, for instance 4ti2 [4t]. In what follows, we will need the following well-known lemma, which follows from the definition of a Markov basis and elementary facts about lattice ideals (see, e.g., (Miller and Sturmfels 2005, Proposition 7.5)).

### Lemma 2.3

Let $\Lambda \subseteq \ker _{\mathbb {Z}}(\textbf{A})$ be a Markov basis for $\textbf{A}\in \mathbb {Z}^{d\times m}$. Then $\Lambda$ is a spanning set of $\ker (\textbf{A})$, and for any ${\varvec{c}}\in \mathbb {R}_{>0}^m$ it holds that

$$\begin{aligned} \overline{\mathcal {X}}_{\textbf{A},{\varvec{c}}}= \left\{ {\varvec{x}}\in \mathbb {R}_{\ge 0}^m : {\varvec{c}}^{{\varvec{\gamma }}^+}{\varvec{x}}^{{\varvec{\gamma }}^-}={\varvec{c}}^{{\varvec{\gamma }}^-}{\varvec{x}}^{{\varvec{\gamma }}^+} \text {for\; all}\,\, {\varvec{\gamma }}\in \Lambda \right\} . \end{aligned}$$

### Example 2.4

The following will appear throughout the paper as a running example. Here, we use it to illustrate the importance of picking a sufficiently large spanning set $\Lambda$ when considering the Euclidean closure $\overline{\mathcal {X}}_{\textbf{A},{\varvec{c}}}$. Let $d=2$ and $m=4$, and consider the log-linear model given by

$$\begin{aligned} \textbf{A}=\begin{pmatrix}4& 2& 3& 1\\ 0& 2& 1& 3\end{pmatrix}. \end{aligned}$$

This defines the set

2.4 $$\begin{aligned} \mathcal {X}_{\textbf{A}}= \left\{ (t_1^4,t_1^2t_2^2,t_1^3t_2,t_1t_2^3):(t_1,t_2)\in \mathbb {R}_{>0}^2\right\} . \end{aligned}$$

An example of a Markov basis for $\textbf{A}$ is

$$\begin{aligned} \Lambda _\textrm{mb}=\{(1,1,-2,0),\,(0,2,-1,-1),\, (2,0,-3,1),\, (1,-1,-1,1)\}, \end{aligned}$$

and it follows from Lemma 2.3 that

$$\begin{aligned} \overline{\mathcal {X}}_{\textbf{A}}= \{(x_1,x_2,x_3,x_4)\in \mathbb {R}_{\ge 0}^4: x_1x_2=x_3^2,\, x_2^2=x_3x_4,\, x_1^2x_4=x_3^3,\, x_1x_4=x_2x_3 \}. \end{aligned}$$

On the other hand, the vector space basis

$$\begin{aligned} \Lambda _1=\{(1,1,-2,0), (0,2,-1,-1)\} \end{aligned}$$

is not a Markov basis, and the resulting binomials cut out a strictly larger set in $\mathbb {R}_{\ge 0}^4$:

$$\begin{aligned} \{(x_1,x_2,x_3,x_4)\in \mathbb {R}_{\ge 0}^4: x_1x_2=x_3^2, \,x_2^2=x_3x_4 \}=\overline{\mathcal {X}}_{\textbf{A}}\cup \{(0,x_2,x_3,0): x_2,x_3\in \mathbb {R}_{\ge 0}\}. \end{aligned}$$

In contrast, it follows from (2.3) that the restriction to the *positive* orthant $\mathbb {R}_{>0}^4$ can be described by either spanning set:

$$\begin{aligned} \mathcal {X}_{\textbf{A}}&= \{ (x_1,x_2,x_3,x_4)\in \mathbb {R}_{>0}^4 : x_1x_2=x_3^2,\, x_2^2=x_3x_4,\, x_1^2x_4=x_3^3,\,x_1x_4 = x_2x_3 \} \\&= \{(x_1,x_2,x_3,x_4)\in \mathbb {R}_{>0}^4 : x_1x_2=x_3^2,\, x_2^2=x_3x_4\}. \end{aligned}$$

Thus far, we have focused on parametric and implicit descriptions of log-affine models. Now we turn to *maximum likelihood estimation*. Suppose we observe independently and identically distributed data ${\varvec{u}}=(u_1,\ldots ,u_m)\in \mathbb {R}_{\ge 0}^m{\setminus }\{{\varvec{0}}\}$ where $u_j$ is the number of observations of state *j*. We denote the total number of observations by $u_+=\sum _{j=1}^m u_j$, and the corresponding observed distribution by $\bar{{\varvec{u}}}={\varvec{u}}/u_+\in \Delta _{m-1}$. A maximum likelihood estimate (MLE) of ${\varvec{u}}$ for a model $\mathcal {M}\subseteq \Delta _{m-1}$ is a distribution $\widehat{{\varvec{p}}}\in \mathcal {M}$ that “best explains” the data, in the sense that it maximizes the likelihood function

$$\begin{aligned} L_{{\varvec{u}}} :\mathcal {M}\rightarrow \mathbb {R},\quad {\varvec{p}}\mapsto p_1^{u_1} \dots p_m^{u_m}. \end{aligned}$$

For log-linear models, there is a well-known characterization of the (unique) MLE as the point in the model that satisfies some linear constraints. This will later be the foundation for the construction in Sect. 4. In the algebraic statistics literature, this and similar results are often referred to as Birch’s Theorem (Pachter and Sturmfels 2005, Sect. 1.2). For proofs of the version we give here, see (Drton et al. 2008, Proposition 2.1.5), (Sullivant 2018, Theorem 8.2.1), and (Lauritzen 1996, Theorem 4.8).

### Theorem 2.5

(Birch’s Theorem). Let $\textbf{A} \in \mathbb {Z}^{d \times m}$ with $\mathbbm {1}\in {{\,\textrm{row}\,}}(\textbf{A})$, let ${\varvec{c}} \in \mathbb {R}_{>0}^m$, and let ${\varvec{u}}\in \mathbb {R}_{\ge 0}^m$ be a vector such that $\bar{{\varvec{u}}}+\ker (\textbf{A})$ intersects $\mathbb {R}_{>0}^m$. Then there is a unique solution to the system

$$\begin{aligned} \widehat{{\varvec{p}}} \in \overline{\mathcal {X}}_{\textbf{A},{\varvec{c}}}\quad \text {and}\quad {\textbf{A}} \widehat{{\varvec{p}}}={\textbf{A}} \bar{{\varvec{u}}}. \end{aligned}$$

This solution $\widehat{{\varvec{p}}}$ has positive entries, and constitutes the MLE of ${\varvec{u}}$ for the model $\mathcal {M}_{\textbf{A},{\varvec{c}}}$.

From the discussion above, it is clear that the MLE is an algebraic function of the observed data. Interestingly, it is sometimes a rational function of ${\varvec{u}}$, which the following example illustrates. Determining which log-affine models have this property and how the rational MLE depends on $\textbf{A}$ and ${\varvec{c}}$ is a central topic in algebraic statistics, and has been studied in, e.g., (Huh 2014; Duarte et al. 2021; Davies et al. 2023).

### Example 2.6

An additional example, which we will use for figures and explicit calculations throughout the paper, is the Hardy–Weinberg model, given by

$$\begin{aligned} \textbf{A}=\begin{pmatrix}0 & 1 & 2\\ 2 & 1 & 0\end{pmatrix}\quad \text {and}\quad {\varvec{c}}=(1,2,1). \end{aligned}$$

The name alludes to the fact that this model appears in population genetics: each point $(t_1^2,2t_1t_2,t_2^2)\in \mathcal {M}_{\textbf{A},{\varvec{c}}}$ encodes the distribution of the three possible diploid genotypes for a gene with two alleles with frequencies $t_1$ and $t_2$. The MLE is a rational function of the observed data ${\varvec{u}}\in \mathbb {Z}_{>0}^m$ (see, e.g., (Huh and Sturmfels 2014, Example 1.3)):

2.5 $$\begin{aligned} \widehat{{\varvec{p}}}= \left( \frac{(2u_1+u_2)^2}{4(u_1+u_2+u_3)^2} , \frac{(u_2+2u_3)(2u_1+u_2)}{2(u_1+u_2+u_3)^2} , \frac{(u_2+2u_3)^2}{4(u_1+u_2+u_3)^2} \right) . \end{aligned}$$

**Fig. 1:**
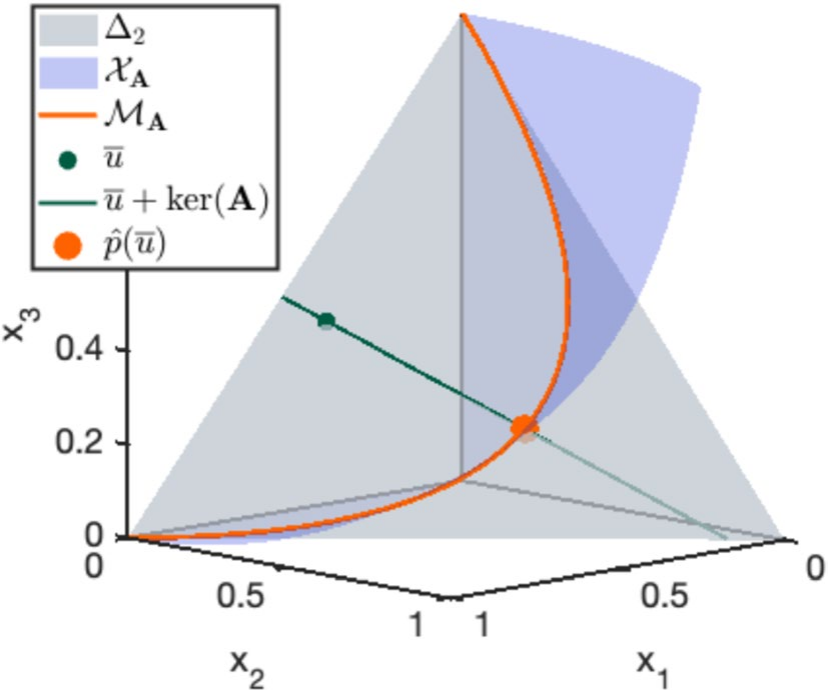
Illustration of Birch’s Theorem for the Hardy–Weinberg model (Example 2.6). The model $\mathcal {M}_{\textbf{A}}$ (orange) is the intersection of $\mathcal {X}_{\textbf{A},{\varvec{c}}}$ (blue) and the probability simplex $\Delta _2$ (gray). Birch’s Theorem guarantees that for any observed distribution $\bar{{\varvec{u}}}\in \Delta _{2}$, there is a unique point $\widehat{{\varvec{p}}}$ in the intersection between $\overline{\mathcal {X}}_{\textbf{A}}$ and $\bar{{\varvec{u}}} + \ker (\textbf{A})$ (green), and that this is the MLE

The model, the positive toric variety, and the MLE are illustrated in Figure 1.

We end the section by introducing two more examples of models that play an important role in the algebraic statistics literature, and which we will use for our numerical experiments in Sect. 5.3. For the first model, the MLE depends rationally on the observed data ${\varvec{u}}$, whereas this is not the case for the other model.

### Example 2.7

Given two random variables with $r_1$ and $r_2$ states, respectively, their joint distribution is a point ${\varvec{p}}=(p_{ij})_{(i,j)\in [r_1]\times [r_2]}\in \Delta _{r_1r_2-1}$, where $p_{ij}$ is the probability that the first random variable is in state *i* and the second is in state *j*. If the two random variables are independent, the joint distribution belongs to the independence model, which is the image of the parametrization

$$\begin{aligned} \Delta _{r_1-1}\times \Delta _{r_2-1}\rightarrow \Delta _{r_1r_2-1}, \quad ((a_1,\dots , a_{r_1}), (b_1,\dots , b_{r_2}))\mapsto (a_ib_j)_{(i,j)\in [r_1]\times [r_2]}. \end{aligned}$$

This is a log-linear model, corresponding to the design matrix

$$\begin{aligned} \textbf{A}_\textrm{ind}(r_1,r_2){:}{=}\left( \begin{array}{ccc ccc c ccc} 1 & \cdots & 1 \\ & & & 1 & \cdots & 1 \\ & & & & & & \ddots \\ & & & & & & & 1 & \cdots & 1 \\ \hline \\ 1 & & & 1 & & & & 1 & & \\ & \ddots & & & \ddots & & \cdots & & \ddots & \\ & & 1 & & & 1 & & & & 1 \\ \end{array}\right) \in \mathbb {Z}^{(r_1+r_2)\times r_1r_2}. \end{aligned}$$

This model has a unique minimal Markov basis (Drton et al. 2008, Proposition 1.2.2) given by

2.6 $$\begin{aligned} \pm ( {\varvec{e}}_{ij}+{\varvec{e}}_{k\ell }-{\varvec{e}}_{i\ell }-{\varvec{e}}_{kj}) , \quad \text { for }\, 1 \le i< k \le r_1,\,\, 1 \le j < \ell \le r_2 \end{aligned}$$

where ${\varvec{e}}_{ij}$ is the standard basis vector of $\mathbb {R}^{r_1r_2}$ corresponding to the $p_{ij}$ coordinate. It can be verified that the following rational expression in ${\varvec{u}}$ satisfies the conditions of Birch’s Theorem, and therefore is the MLE:

2.7 $$\begin{aligned} \widehat{p}_{ij}=\frac{\big (\sum _{\ell =1}^{r_2} u_{i\ell }\big )\big (\sum _{k=1}^{r_1} u_{kj}\big )}{\big (\sum _{k=1}^{r_1}\sum _{\ell =1}^{r_2} u_{k\ell }\big )^2}. \end{aligned}$$

### Example 2.8

The theory of graphical models is a rich source of examples of log-linear models. A particularly interesting example is the undirected 4-cycle (Geiger et al. 2006, Example 4), which has a $16\times 16$ design matrix, and is the smallest graphical model for which the MLE does not depend rationally on the observed distribution.

## Reaction network theory: detailed-balanced systems

Reaction network theory is the study of certain families of parametrized systems of ordinary differential equations (ODEs) encoded by directed graphs. In this section, we review some basic notions in reaction network theory, including the notion of *detailed-balanced* steady states. For a more comprehensive introduction to the field, see the review articles (Gunawardena 2003; Dickenstein 2016; Yu and Craciun 2018; Feliu and Shiu 2025), and the textbook (Feinberg 2019).

### Definition 3.1

A reaction network with species $\{\textsf {X}_1,\ldots ,\textsf {X}_m\}$ is a simple directed graph $G = (V,E)$ with no isolated vertices, where the vertices are points in $\mathbb {Z}_{\ge 0}^m$ (interpreted as formal linear combinations of the species), referred to as complexes, and the edges, denoted ${\varvec{y}}_i \rightarrow {\varvec{y}}_j$ for ${\varvec{y}}_i,{\varvec{y}}_j\in V$, are referred to as reactions of the network. A reaction network *G* is said to be reversible if every reaction is part of a reversible pair, i.e., ${\varvec{y}}_i \rightarrow {\varvec{y}}_j \in E$ if and only if ${\varvec{y}}_j \rightarrow {\varvec{y}}_i \in E$. We write ${\varvec{y}}_i \rightleftharpoons {\varvec{y}}_j$ for such a reaction pair.

The goal of reaction network theory is to model the time evolution of the concentrations ${\varvec{x}}=(x_1,\ldots ,x_m)$ of the species $\textsf {X}_1,\ldots ,\textsf {X}_m$ of the network under some kinetic assumptions. One of the most common, *mass-action kinetics*, is based on the first principle that the reaction rate is proportional to the collision probability of the reacting species.

### Definition 3.2

A mass-action system is a weighted directed graph $(G,{\varvec{{k}}})$, where *G* is a reaction network, and ${\varvec{{k}}}= ({k}_{ij})_{{\varvec{y}}_i\rightarrow {\varvec{y}}_j\in E}$ is a tuple of positive real numbers called rate constants, indexed by the reactions. The induced dynamics of a mass-action system $(G,{\varvec{k}})$ is given by the ODEs

3.1 $$\begin{aligned} \frac{d{\varvec{x}}}{dt} = \sum _{{\varvec{y}}_i\rightarrow {\varvec{y}}_j\in E} {k}_{ij} {\varvec{x}}^{{\varvec{y}}_i} ({\varvec{y}}_j - {\varvec{y}}_i), \end{aligned}$$

where the right-hand side is a vector of polynomials that we will denote ${\varvec{f}}_{G,{\varvec{k}}}({\varvec{x}})$. For an initial value ${\varvec{x}}_0\in \mathbb {R}_{\ge 0}^m$, we denote the corresponding trajectory of (3.1) by ${\varvec{x}}(t;{\varvec{x}}_0)$ or simply ${\varvec{x}}(t)$.

The nonnegative and positive orthants are *forward-invariant* with respect to (3.1) (Aizik 1972; Eduardo 2001). In other words, if ${\varvec{x}}_0\in \mathbb {R}_{\ge 0}^m$, then ${\varvec{x}}(t;{\varvec{x}}_0)\in \mathbb {R}_{\ge 0}^m$ for all $t\ge 0$ for which the trajectory is defined, and, analogously, if ${\varvec{x}}_0\in \mathbb {R}_{>0}^m$, then ${\varvec{x}}(t;{\varvec{x}}_0)\in \mathbb {R}_{>0}^m$ for all $t\ge 0$ for which the trajectory is defined.

The stoichiometric subspace of a reaction network *G* is the linear subspace

$$\begin{aligned} S {:}{=}{{\,\textrm{span}\,}}_\mathbb {R}\{ {\varvec{y}}_j - {\varvec{y}}_i: {\varvec{y}}_i \rightarrow {\varvec{y}}_j \in E\}\subseteq \mathbb {R}^m. \end{aligned}$$

Each initial value ${\varvec{x}}_0$ defines a stoichiometric compatibility class

$$\begin{aligned} ({\varvec{x}}_0+S)_{\ge 0} {:}{=}\{{\varvec{x}}_0+{\varvec{s}}: {\varvec{s}}\in S \}\cap \mathbb {R}_{\ge 0}^m, \end{aligned}$$

where the part intersecting the positive orthant will be denoted $({\varvec{x}}_0+S)_{>0}$. Since the right-hand side of (3.1) lies in *S* for any ${\varvec{x}}\in \mathbb {R}_{>0}^m$, the subset $({\varvec{x}}_0+S)_{>0}$ is also forward-invariant. If $\dim (S) < m$, the nontrivial stoichiometric compatibility classes are given by conservation laws, which are linear first integrals, represented by a linear system $\textbf{W} {\varvec{x}}= \textbf{W} {\varvec{x}}_0$, where the rows of $\textbf{W}$ span $S^\perp$, and ${\varvec{x}}_0$ is the initial state. The linear forms $\textbf{W} {\varvec{x}}$ are referred to as conserved quantities, and $\textbf{W} {\varvec{x}}_0$ as total amounts.

### Example 3.3

Consider a reaction network consisting of two reversible reactions:Its associated ODE system is

$$\begin{aligned} \frac{d{\varvec{x}}}{dt} = \left( \begin{array}{l} k_{12}x_3^2 - k_{21} x_1x_2\\ k_{12}x_3^2 - k_{21} x_1x_2 + 2k_{34}x_3x_4 - 2k_{43}x_2^2 \\ -2k_{12}x_3^2 + 2k_{21} x_1x_2 - k_{34}x_3x_4 + k_{43}x_2^2 \\ -k_{34}x_3x_4 + k_{43} x_2^2 \end{array}\right) . \end{aligned}$$

Its stoichiometric subspace *S* is the two-dimensional subspace spanned by $(1,1,-2,0)$ and $(0,2,-1,-1)$. Two conservation laws are expected since ${{\,\textrm{codim}\,}}(S) = 2$; for example,

$$\begin{aligned} \begin{pmatrix} 4 & 2 & 3 & 1 \\ 0 & 2 & 1 & 3 \end{pmatrix} ( {\varvec{x}} - {\varvec{x}}_0) = {\varvec{0}}, \end{aligned}$$

as the rows of the matrix span $S^\perp$. One can also confirm the conservation laws by checking that $4\dot{x}_1 + 2\dot{x}_2 + 3 \dot{x}_3 + \dot{x}_4= 0$ and $2 \dot{x}_2 + \dot{x}_3 + 3\dot{x}_4 = 0$. The choice of conservation laws is not unique; for example one can also check that $\dot{x}_1 + \dot{x}_2 + \dot{x}_3 + \dot{x}_4 = 0$.

Mass-action systems are capable of very diverse dynamics; they can display oscillation, have multiple stable steady states in the same stoichiometric compatibility class, and even exhibit chaotic dynamics (e.g., (Gáspár and Tóth 2023, Fig. 21)). That said, some classes of mass-action systems are known to be particularly stable. The most well-known are *detailed-balanced systems*, which originated from Boltzmann (1887, 1896).

### Definition 3.4

Let $(G,{\varvec{{k}}})$ be a mass-action system in $\mathbb {R}^m$ with associated ODEs $\dot{{\varvec{x}}} = {\varvec{f}}_{G,{\varvec{{k}}}}({\varvec{x}})$.

- A point $\widehat{{\varvec{x}}} \in \mathbb {R}_{\ge 0}^m$ is a steady state if ${\varvec{f}}_{G,{\varvec{{k}}}}(\widehat{{\varvec{x}}}) = {\varvec{0}}$. It is a positive steady state if $\widehat{{\varvec{x}}} \in \mathbb {R}_{>0}^m$; otherwise it is a boundary steady state. The set of all positive steady states is denoted $\mathcal {E}_{G,{\varvec{{k}}}}$.
- If the network *G* is reversible, then a positive steady state $\widehat{{\varvec{x}}}$ is detailed-balanced if
  3.3 $$\begin{aligned} {k}_{ij}\,\widehat{{\varvec{x}}}^{{\varvec{y}}_i}={k}_{ji}\, \widehat{{\varvec{x}}}^{{\varvec{y}}_j} \text { for all } {\varvec{y}}_i\rightleftharpoons {\varvec{y}}_j. \end{aligned}$$
  The set of positive detailed-balanced steady states is denoted $\mathcal {D}_{G, {\varvec{{k}}}}$.

If a mass-action system $(G,{\varvec{k}})$ has a positive steady state that is detailed-balanced, then all positive steady states are detailed-balanced; therefore, we say $(G,{\varvec{{k}}})$ is a detailed-balanced mass-action system if $\mathcal {D}_{G,{\varvec{{k}}}}$ is nonempty. This, and other key properties of detailed-balanced systems, are collected in the following theorem. We note that these results were originally proved for the more general class of *complex-balanced* mass-action systems (see Horn and Jackson 1972 for a definition and precise statements).

### Theorem 3.5

(Horn and Jackson 1972). Let $(G,{\varvec{{k}}})$ be a reversible mass-action system with stoichiometric subspace *S*, and suppose there exists a detailed-balanced steady state $\widehat{{\varvec{x}}} \in \mathcal {D}_{G,{\varvec{{k}}}}$. Then the following holds:

- $$\mathcal {D}_{G,{\varvec{{k}}}} = \mathcal {E}_{G,{\varvec{{k}}}}$$.
- The set of positive steady states is given by
  $$\begin{aligned} \mathcal {E}_{G,{\varvec{{k}}}} = \{ {\varvec{x}}\in \mathbb {R}_{>0}^m: \log {\varvec{x}}- \log \widehat{{\varvec{x}}} \in S^\perp \}=\{\widehat{{\varvec{x}}} \circ {\varvec{t}}^{\textbf{W}}: {\varvec{t}}\in \mathbb {R}_{>0}^{d}\} \end{aligned}$$
  for any matrix $\textbf{W}\in \mathbb {R}^{d\times m}$ whose rows span $S^\perp$.
- There is exactly one steady state in every positive stoichiometric compatibility class $({\varvec{x}}_0 + S)_{>0}$.
- The unique positive steady state in $({\varvec{x}}_0+S)_{>0 }$ is asymptotically stable within $({\varvec{x}}_0 + S)_{>0}$.

In the language of Sect. 2, part (b) says that $\mathcal {E}_{G,{\varvec{k}}}=\mathcal {X}_{\textbf{W},\widehat{{\varvec{x}}}}$, and part (c) corresponds to Birch’s Theorem (Theorem 2.5), with the observed distribution playing the role of ${\varvec{x}}_0$ and the MLE playing the role of the detailed-balanced steady state $\widehat{{\varvec{x}}}$.

### Remark 3.6

The strictly convex function $V({\varvec{x}}) = {\varvec{x}}{}\cdot (\log {\varvec{x}}- \log {\varvec{x}}^* - \mathbbm {1})$, known as the free energy or entropy function in thermodynamics, is a Lyapunov function defined on $\mathbb {R}_{>0}^m$ for the system (3.1), with global minimum at ${\varvec{x}}^*$. The similarity to the entropy of a discrete distribution foreshadows the correspondence between positive steady states and MLEs spelled out in Sect. 4; while the former minimize *V*, the latter minimize the *Kullback–Leibler divergence* $\operatorname {KL}(\bar{{\varvec{u}}} || {\varvec{p}})$ to the empirical distribution $\bar{{\varvec{u}}}$. See (Améndola et al. 2021, Sect. 2) and (Craciun et al. 2009, Proposition 11).

### Example 3.7

Consider the following network with a single reversible reaction pair:The corresponding mass-action system is

$$\begin{aligned} \dot{x}_1 = x_2^2-4x_1x_3,\quad \dot{x}_2= -2x_2^2+8x_1x_3,\quad \dot{x}_3=x_2^2-4x_1x_3, \end{aligned}$$

and two linearly independent conserved quantities are given by the rows of the matrix

$$\begin{aligned} \textbf{W}=\begin{pmatrix}2 & 1 & 0\\ 0 & 1 & 2\end{pmatrix}. \end{aligned}$$

The system is detailed-balanced, where

$$\begin{aligned} \mathcal {E}_{G,{\varvec{k}}}=\mathcal {D}_{G,{\varvec{k}}}=\{(t_1^2,2t_1t_2,t_2^2):(t_1,t_2)\in \mathbb {R}_{>0}^2\}\subseteq \mathbb {R}_{>0}^3. \end{aligned}$$

**Fig. 2:**
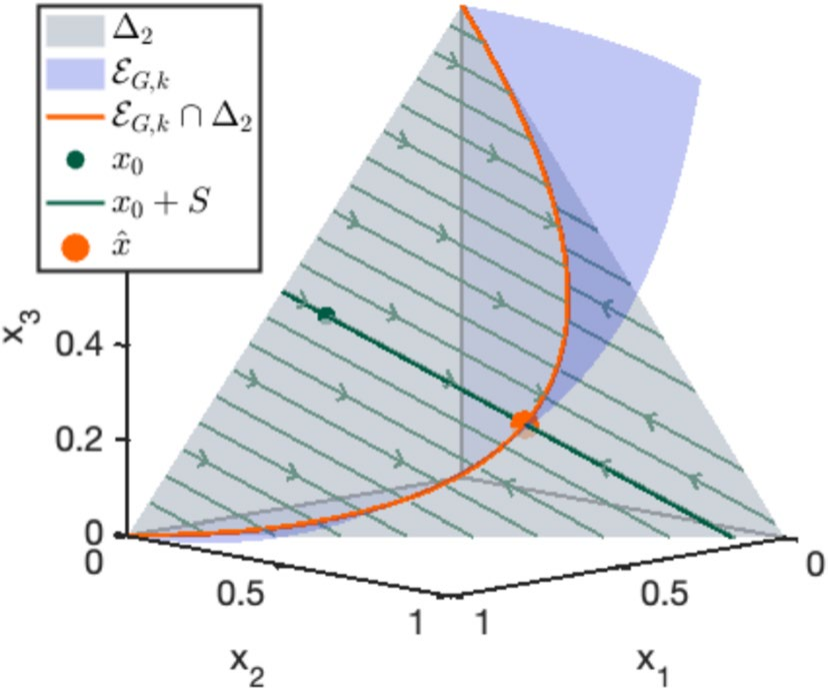
Illustration of Example 3.7, showing the set of positive steady states $\mathcal {E}_{G,{\varvec{k}}}$ (blue), an initial state ${\varvec{x}}_0$ and the corresponding stoichiometric compatibility class ${\varvec{x}}_0+S$ (green), and the unique positive steady state $\widehat{{\varvec{x}}}$ (orange), and the phase portrait restricted to $\Delta _2$ (light green). Note that $\dim (S) = 1$

Figure 2 shows the phase portrait restricted to $\Delta _2$, a stoichiometric compatibility class with an initial state ${\varvec{x}}_0$ and the corresponding steady state $\hat{{\varvec{x}}}$. The similarity with Figure 1 is not a coincidence; we will see in Sect. 4 that this network is obtained by Construction 4.1 for the MLE problem of Example 2.6.

An important property that we consider in the next section is *global convergence*, and we therefore devote the remainder of this section to discussing global convergence properties of detailed-balanced systems.

A long-standing conjecture in reaction network theory is that the unique positive steady state in each stoichiometric compatibility class is not only locally stable, but *globally stable* (Horn 1974). This conjecture is referred to as the *Global Attractor Conjecture*, and has a rich history (see Craciun et al. 2013; Craciun 2019). It has been proven for several classes of networks, including networks where the underlying graph is strongly connected (David 2011; Boros and Hofbauer 2020); other proven cases involve graph-theoretic and geometric assumptions on the network, see, e.g., (Pantea 2012; Craciun et al. 2013; Gopalkrishnan et al. 2014).

It has been proven that if a positive steady state of a complex-balanced system fails to be a global attractor, then there exist trajectories that approach the boundary (Siegel and MacLean 2000; Angeli et al. 2007; Shiu and Sturmfels 2010). Below, we state this result in the special case of detailed-balanced systems. Recall that the $\omega$-limit set of a trajectory ${\varvec{x}}(t; {\varvec{x}}_0)$ is the set of subsequential limit points, i.e.,

$$\begin{aligned} \omega ({\varvec{x}}_0) = \left\{ {\varvec{y}}\in \mathbb {R}^n:{\varvec{y}}=\lim _{n\rightarrow \infty }{\varvec{x}}(t_n;{\varvec{x}}_0) \text { for some increasing sequence }(t_n)_{n=1}^\infty \right\} . \end{aligned}$$

### Proposition 3.8

(Siegel and MacLean 2000, Theorem 3.2). Let $(G,{\varvec{{k}}})$ be a detailed-balanced mass-action system, and let ${\varvec{x}}_0 \in \mathbb {R}_{>0}^m$. Then the $\omega$-limit set $\omega ({\varvec{x}}_0)$ either consists of boundary steady states or the unique positive steady state in $({\varvec{x}}_0 +S)_{>0}$.

A network is said to lack relevant boundary steady states if no stoichiometric compatibility class that intersects the positive orthant contains a steady state on the boundary of the nonnegative orthant. A well-known consequence of Proposition 3.8 is that this property implies global convergence.

### Proposition 3.9

(Siegel and MacLean 2000). Let $(G,{\varvec{k}})$ be a detailed-balanced mass-action system that lacks relevant boundary steady states. Then for any ${\varvec{x}}_0\in \mathbb {R}_{>0}^m$, the trajectory ${\varvec{x}}(t;{\varvec{x}}_0)$ converges to the unique positive steady state $\widehat{{\varvec{x}}}$ in $\mathcal {E}_{G,{\varvec{k}}}\cap ({\varvec{x}}_0+S)_{>0}$.

A useful condition to rule out relevant boundary steady states comes from the theory of siphons, developed in Angeli et al. (2007) and expanded in several subsequent works, e.g., (Shiu and Sturmfels 2010; Deshpande and Gopalkrishnan 2014; Al-Radhawi and Angeli 2016; Marcondes de Freitas 2017).

### Definition 3.10

A siphon in a reaction network $G = (V,E)$ with species $\{\textsf {X}_1,\ldots ,\textsf {X}_m\}$ is a set of indices $Z\subseteq [m]$ such that for every $i\in Z$ and every reaction ${\varvec{y}}\rightarrow {\varvec{y}}' \in E$ with $y'_i>0$, there exists $j\in Z$ such that $y_j>0$.

Intuitively, a siphon keeps track of which species can simultaneously have zero concentrations at equilibrium. It turns out that there cannot exist relevant boundary steady states if “mass is conserved” within each minimal siphon, in the following sense.

### Proposition 3.11

(Angeli et al. 2007, (Marcondes de Freitas 2017, Proposition 2(v))). Consider a network with stoichiometric subspace $S\subseteq \mathbb {R}^m$. Suppose that for every inclusion-minimal siphon $Z\subseteq [m]$, there exists ${\varvec{w}}\in S^\perp \cap \mathbb {R}_{\ge 0}^m$ with ${{\,\textrm{supp}\,}}({\varvec{w}})=Z$. Then the network lacks relevant boundary steady states.

A network that satisfies the condition in Proposition 3.11 is said to be structurally persistent.

We end this section by using the notion of siphons to extend Theorem 3.5(a) and Proposition 3.9 to also account for the dynamics on the boundary $\partial \mathbb {R}_{\ge 0}^m$, which we will need in Sect. 5.1.

Consider the set of nonnegative steady states

$$\begin{aligned} \mathcal {E}_{G,{\varvec{{k}}}}^{\ge 0} {:}{=}\left\{ {\varvec{x}}\in \mathbb {R}_{\ge 0}^m: {\varvec{f}}_{G,{\varvec{{k}}}}({\varvec{x}}) = {\varvec{0}} \right\} . \end{aligned}$$

We say $\widehat{{\varvec{x}}} \in \mathcal {E}_{G,{\varvec{{k}}}}^{\ge 0}$ is a nonnegative detailed-balanced steady state if it satisfies (3.3); we denote the set of such points by $\mathcal {D}_{G,{\varvec{{k}}}}^{\ge 0}$. The following is the detailed-balanced analog of Cappelletti and Wiuf (2016, Theorem 3.1).

### Proposition 3.12

Let $(G,{\varvec{k}})$ be a detailed balanced mass-action system. Then the set of nonnegative steady states and the set of nonnegative detailed-balanced states coincide, i.e., $\mathcal {E}_{G,{\varvec{k}}}^{\ge 0}=\mathcal {D}_{G,{\varvec{k}}}^{\ge 0}$.

### Proof

The inclusion $\mathcal {D}_{G,{\varvec{k}}}^{\ge 0}\subseteq \mathcal {E}_{G,{\varvec{k}}}^{\ge 0}$ is immediate. To prove the inclusion $\mathcal {E}_{G,{\varvec{k}}}^{\ge 0}\subseteq \mathcal {D}_{G,{\varvec{k}}}^{\ge 0}$, we begin by introducing some notation. For a set $Z\subseteq [m]$, let $G^Z$ denote the subnetwork of *G* obtained by removing all reactions where a species with index in *Z* appears as a reactant, and let ${\varvec{k}}^Z$ be the restriction of ${\varvec{k}}$ to the reactions of $G^Z$. For any vector ${\varvec{x}}\in \mathbb {R}_{\ge 0}^m$ and $Z \subseteq [m]$, we denote by ${\varvec{x}}^Z$ the vector in $\mathbb {R}_{\ge 0}^m$ where $x^Z_i = x_i$ for all $i \not \in Z$, and $x^Z_i = 0$ for all $i \in Z$. Finally, by a slight abuse of notation, we view the sets of positive steady states $\mathcal {E}_{G^{Z},{\varvec{k}}^Z}$ and positive detailed-balanced steady state $\mathcal {D}_{G^Z,{\varvec{k}}^Z}$ of the subnetwork as being embedded in $\mathbb {R}^m$, by supplementing with $\widehat{x}_i = 0$ for any $i\in Z$. Then it holds that

$$\begin{aligned} \mathcal {E}_{G,{\varvec{k}}}^{\ge 0}=\bigcup _{Z\subseteq [m]} \mathcal {E}_{G^Z,{\varvec{k}}^Z}. \end{aligned}$$

We claim that for every $Z\subseteq [m]$ such that $\mathcal {E}_{G^Z,{\varvec{k}}^Z}$ is nonempty, *Z* must be a siphon. Indeed, if *Z* is not a siphon, then $G^Z$ has a reaction where some species $\textsf {X}_i$ with $i\in Z$ is formed, but no species $\textsf {X}_j$ with $j\in Z$ are reactants, which means that this reaction appears in $G^Z$. Since $G^Z$ by construction does not have any reactions where $\textsf {X}_i$ is consumed, this implies that $\mathcal {E}_{G^Z,{\varvec{k}}^Z}$ is empty.

Suppose $Z\subseteq [m]$ is a siphon. Then for every reversible reaction pair ${\varvec{y}}\rightleftharpoons {\varvec{y}}'$ that appears in $G^Z$, it holds that neither ${\varvec{y}}$ nor ${\varvec{y}}'$ involves any species with index in *Z*. Hence, $\widehat{{\varvec{x}}}^Z\in \mathcal {D}_{G^Z,{\varvec{k}}^Z}$ for every $\widehat{{\varvec{x}}}\in \mathcal {D}_{G,{\varvec{k}}}$. This implies that $\mathcal {D}_{G^Z,{\varvec{k}}^Z}$ is nonempty, and therefore equal to $\mathcal {E}_{G^Z,{\varvec{k}}^Z}$ by Theorem 3.5(a). Thus we obtain

$$\begin{aligned} \mathcal {E}^{\ge 0}_{G,{\varvec{{k}}}} = \bigcup _{\begin{array}{c} Z \text { is a} \\ \text {siphon} \end{array}} \mathcal {E}_{G^Z, {\varvec{{k}}}^Z} = \bigcup _{\begin{array}{c} Z \text { is a} \\ \text {siphon} \end{array}} \mathcal {D}_{G^Z, {\varvec{{k}}}^Z} \subseteq \mathcal {D}^{\ge 0}_{G,{\varvec{{k}}}}. \end{aligned}$$

$$\square$$

The following extends Proposition 3.9 to the case when the system is initialized at $\partial \mathbb {R}_{\ge 0}^m$.

### Proposition 3.13

Let $(G,{\varvec{k}})$ be a detailed-balanced mass-action system that lacks relevant boundary steady states, and let ${\varvec{x}}_0\in \mathbb {R}_{\ge 0}^m$ be such that $({\varvec{x}}_0+S)_{>0}$ is nonempty. Then the trajectory ${\varvec{x}}(t;{\varvec{x}}_0)$ converges to the unique positive steady state $\widehat{{\varvec{x}}}$ in $\mathcal {E}_{G,{\varvec{k}}}\cap ({\varvec{x}}_0+S)_{>0}$

### Proof

The desired claim follows from Proposition 3.9 if the trajectory ${\varvec{x}}(t;{\varvec{x}}_0)$ enters the positive orthant at some $t>0$. Suppose for a contradiction that the trajectory is fully contained in $\partial \mathbb {R}_{\ge 0}^m$, and consider the (then nonempty) set

$$\begin{aligned} Z:=\left\{ i\in [m]:x_i(t;{\varvec{x}}_0)=0\,\text { for all }t>0\right\} . \end{aligned}$$

It follows from (Angeli et al. 2007, Proposition 2) that *Z* is a siphon. Then, using the notation in the proof of Proposition 3.12, $(G^Z,{\varvec{k}}^Z)$ is a detailed-balanced subnetwork, with $\widehat{{\varvec{x}}}^Z$ as a steady state. Since, $\widehat{{\varvec{x}}}^Z$ lies in the same stoichiometric compatibility class as ${\varvec{x}}_0={\varvec{x}}_0^Z$, this contradicts the assumption that $(G,{\varvec{k}})$ lacks relevant boundary steady states. $\square$

## Reaction network representation of log-affine models

The goal of this section is to connect the following two scenarios:

- Algebraic statistics: We saw in Theorem 2.5 that for a log-affine model $\mathcal {M}_{\textbf{A},{\varvec{c}}}$, the unique MLE for a vector of counts ${\varvec{u}}\in \mathbb {R}_{>0}^m$ is given by $\mathcal {M}_{\textbf{A},{\varvec{c}}}\cap (\bar{{\varvec{u}}}+\ker (\textbf{A}))$.
- Reaction network theory: We saw in Theorem 3.5 that for a detailed-balanced system with positive steady state set $\mathcal {E}_{G,{\varvec{{k}}}}$ and an initial value ${\varvec{x}}_0 \in \mathbb {R}_{>0}^m$, the unique positive steady state is given by $\mathcal {E}_{G,{\varvec{{k}}}} \cap ({\varvec{x}}_0 + S)$.

More specifically, we will construct a mass-action system for which the set of positive steady states is $\mathcal {X}_{\textbf{A},{\varvec{c}}}$ and the stoichiometric subspace is $S = \ker (\textbf{A})$. Hence, the MLE of the statistical model $\mathcal {M}_{\textbf{A},{\varvec{c}}}$ for an observed distribution $\bar{{\varvec{u}}}$ will be the unique steady state in $(\bar{{\varvec{u}}} + S)_{>0}$.

A key step in the construction will be to choose a finite set $\Lambda \subseteq \ker _{\mathbb {Z}}(\textbf{A})$ that spans $\ker (\textbf{A})$ in the vector space sense. This generalizes the construction in Gopalkrishnan (Gopalkrishnan 2016, Definition 8), which restricts to the case when $\Lambda$ is a $\mathbb {Z}$-basis for $\ker (\textbf{A})$ and ${\varvec{c}}=\mathbbm {1}$. Recall that ${\varvec{\gamma }}^+$ and ${\varvec{\gamma }}^-$ are respectively the nonnegative and nonpositive parts of a vector ${\varvec{\gamma }}\in \mathbb {Z}^m$.

### Construction 4.1

For a finite set of integer vectors $\Lambda \subseteq \mathbb {Z}^m$ that span $\ker (\textbf{A})$, and a vector ${\varvec{c}}\in \mathbb {R}_{>0}^m$, we define the mass-action system $G_{\Lambda , {\varvec{c}}}$ to be the collectionand denote by $G_{\Lambda }$ the underlying reaction network. The associated system of ODEs for $G_{\Lambda ,{\varvec{c}}}$ is

$$\begin{aligned} \frac{d{\varvec{x}}}{dt} = \sum _{{\varvec{\gamma }}\in \Lambda } \left( {\varvec{c}}^{{\varvec{\gamma }}^+} {\varvec{x}}^{{\varvec{\gamma }}^-} - {\varvec{c}}^{{\varvec{\gamma }}^-} {\varvec{x}}^{{\varvec{\gamma }}^+} \right) {\varvec{\gamma }}. \end{aligned}$$

We begin by showing that the set of positive steady states of $G_{\Lambda ,{\varvec{c}}}$ is $\mathcal {X}_{\textbf{A},{\varvec{c}}}$, defined in (2.2).

### Theorem 4.2

Let $\textbf{A} \in \mathbb {Z}^{d\times m}$ with $\mathbbm {1}\in {{\,\textrm{row}\,}}(A)$, and ${\varvec{c}} \in \mathbb {R}_{>0}^m$, and let $\Lambda \subseteq \ker _\mathbb {Z}(\textbf{A})$ be a finite spanning set for $\ker (\textbf{A})$. Then the mass-action system $G_{\Lambda ,{\varvec{c}}}$ in Construction 4.1 is detailed-balanced, and the set of positive steady states is $\mathcal {X}_{\textbf{A},{\varvec{c}}}$.

### Proof

The network $G_\Lambda$ is reversible by construction, and the defining condition (3.3) for detailed-balanced steady states is ${\varvec{c}}^{{\varvec{\gamma }}^+}{\varvec{x}}^{{\varvec{\gamma }}^-}={\varvec{c}}^{{\varvec{\gamma }}^-}{\varvec{x}}^{{\varvec{\gamma }}^+}$ for all ${\varvec{\gamma }}\in \Lambda$. Hence, the the set of detailed-balanced steady states is $\mathcal {X}_{\textbf{A},{\varvec{c}}}$ by (2.3). Since $\mathcal {X}_{\textbf{A},{\varvec{c}}}$ is nonempty, it follows that $G_{\Lambda ,{\varvec{c}}}$ is detailed-balanced, and $\mathcal {X}_{\textbf{A},{\varvec{c}}}$ is the set of all positive steady states by Theorem 3.5(a). $\square$

### Remark 4.3

By construction, the rows of $\textbf{A}$ form a spanning set for $S^\perp$, and hence, they describe conservation laws (i.e., we can take $\textbf{W}=\textbf{A}$ in the notation from Sect. 3), with $\textbf{A}{\varvec{u}}$ being the total amounts. In statistics, these total amounts are commonly referred to as the *sufficient statistics* of the observed data, where the adjective *sufficient* comes from the fact that to compute the MLE for a given ${\varvec{u}}$, it is enough to know the value of $\textbf{A}{\varvec{u}}$.

An important goal of Sect. 5 will be to study how various dynamical properties of the resulting mass-action system depend on the choice of the spanning set $\Lambda$, with particular focus on vector space bases and Markov bases. With the following example, we highlight how one can obtain the same MLE but with different reaction networks and dynamics based on different choices of $\Lambda$.

### Example 4.4

Consider the log-linear model of Example 2.4, given by

$$\begin{aligned} \textbf{A}=\begin{pmatrix}4& 2& 3& 1\\ 0& 2& 1& 3\end{pmatrix}. \end{aligned}$$

- Consider the spanning set $\Lambda _1=\{(1,1,-2,0), (0,2,-1,-1)\}$. The mass-action system $G_{\Lambda _1}$ given by Construction 4.1 is (3.2) of Example 3.3, where all the rate constants are 1 since ${\varvec{c}} = \mathbbm {1}$, and so ${\varvec{c}}^{{\varvec{\gamma }}^+} ={\varvec{c}}^{{\varvec{\gamma }}^-} = 1$.
- In contrast, the Markov basis $\Lambda _\textrm{mb} = \{(1,1,-2,0)$, $(0,2,-1,-1)$, $(1,-1,-1,1)$, $(2,0,-3,1)\}$ generates the mass-action system $G_{\Lambda _\textrm{mb}}$ where all rate constants are 1: 4.1 The top two reactions make up $G_{\Lambda _1}$, so the right-hand side of the associated ODEs of $G_{\Lambda _\textrm{mb}}$ can be written as the sum 
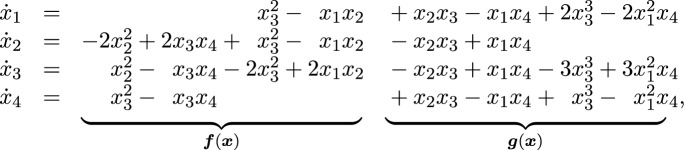
  where $\dot{{\varvec{x}}} = {\varvec{f}}({\varvec{x}})$ is the ODEs associated to $G_{\Lambda _1}$.
- Consider the vector space basis $\Lambda _2 = \{(-4,0,6,-2), \, (3,3,-6,0)\}$. Using this as a spanning set in Construction 4.1, gives the following mass-action system where all rate constants are 1:  This is associated to yet again a different system of ODEs:
  $$\begin{aligned} \dot{x}_1 &= - 4x_1^4x_4^2 + 7 x_3^6 -3 x_1^3 x_2^3,\\ \dot{x}_2 &= 3 x_3^6 -3x_1^3 x_2^3,\\ \dot{x}_3 &= 6x_1^4 x_4^2 - 12 x_3^6 + 6 x_1^3 x_2^3,\\ \dot{x}_4 &= - 2x_1^4x_4^2 + 2x_3^6.\end{aligned}$$

We emphasize that despite the fact that these three networks have different transient dynamics, the sets of positive steady states for all these systems are precisely $\mathcal {X}_{\textbf{A}}$ in (2.4) by Theorem 4.2.

### Remark 4.5

Construction 4.1 is not the only approach to constructing a network with $\mathcal {X}_{\textbf{A},{\varvec{c}}}$ as its positive steady states. For instance, one can decompose each vector ${\varvec{\gamma }}$ into any difference of nonnegative vectors (not just ${\varvec{\gamma }}^+$ and ${\varvec{\gamma }}^-$). Continuing with Example 4.4, we could use $\Lambda _3 = \Lambda _2 \cup \{{\varvec{\mu }}\}$, where ${\varvec{\mu }}= (4,0,0,2) - (3,3,0,0)$. This decomposition gives rise to the network

The following is one of our main results, which states that the MLEs over the model $\mathcal {M}_{\textbf{A},{\varvec{c}}}$ are realized as steady states of the mass-action systems from Construction 4.1.

### Theorem 4.6

Let $\mathcal {M}_{\textbf{A},{\varvec{c}}}$ be the log-affine model defined by $\textbf{A}\in \mathbb {Z}^{d\times m}$ with $\mathbbm {1}\in {{\,\textrm{row}\,}}(\textbf{A})$, and ${\varvec{c}} \in \mathbb {R}_{>0}^m$. Let ${\varvec{u}}\in \mathbb {R}_{\ge 0}^m\setminus \{{\varvec{0}}\}$, and $\bar{{\varvec{u}}} = {\varvec{u}} /u_+$, and suppose $(\bar{{\varvec{u}}}+\ker (\textbf{A}))_{>0}$ is nonempty. Let $\Lambda \subseteq \ker _\mathbb {Z}(\textbf{A})$ be a finite spanning set for $\ker (\textbf{A})$, and let $G_{\Lambda , {\varvec{c}}}$ be the mass-action system in Construction 4.1. Then the MLE $\widehat{{\varvec{p}}}$ of ${\varvec{u}}$ in $\mathcal {M}_{\textbf{A},{\varvec{c}}}$ is the unique steady state of $G_{\Lambda ,{\varvec{c}}}$ in the positive stoichiometric compatibility class $(\bar{{\varvec{u}}}+S)_{>0}$. Furthermore, $\widehat{{\varvec{p}}}$ is asymptotically stable.

### Proof

By Theorem 4.2, the system $G_{\Lambda ,{\varvec{c}}}$ is detailed-balanced, and the positive steady states are given by $\mathcal {X}_{\textbf{A},{\varvec{c}}}$. The positive stoichiometric compatibility class of $\bar{{\varvec{u}}}$ is $(\bar{{\varvec{u}}}+\ker (\textbf{A}))_{>0}$ by Remark 4.3, so the unique steady state in this class is precisely the MLE $\widehat{{\varvec{p}}}$ by Theorem 2.5. Asymptotic stability follows by Theorem 3.5(d). $\square$

### Example 4.7

(The Hardy–Weinberg model revisited). Consider the model from Example 2.6. Using the Markov basis $\Lambda =\{(1,-2,1)\}$, we obtain precisely the mass-action system that appeared in Example 3.7. For a given initial state ${\varvec{x}}_0=(u_1,u_2,u_3)$, the initial value problem has the closed form solution

$$\begin{aligned} x_1(t)&= \frac{ (2u_1+u_2)^2 - (u_2^2-4u_1u_3)e^{-4t} }{4(u_1+u_2+u_3)^2}\\ x_2(t)&= \frac{ (u_2+2u_3)(2u_1+u_2) + (u_2^2-4u_1u_3)e^{-4t}}{2(u_1+u_2+u_3)^2} \\ x_3(t)&= \frac{ (u_2+2u_3)^2 - (u_2^2-4u_1u_3)e^{-4t} }{4(u_1+u_2+u_3)^2}. \end{aligned}$$

Letting $t \rightarrow \infty$, we recover the rational expression for the MLE from (2.5):

$$\begin{aligned} \lim _{t\rightarrow \infty } {\varvec{x}}(t) = \left( \frac{(2u_1+u_2)^2}{4(u_1+u_2+u_3)^2}, \frac{(u_2+2u_3)(2u_1+u_2)}{2(u_1+u_2+u_3)^2}, \frac{(u_2+2u_3)^2}{4(u_1+u_2+u_3)^2} \right) = \widehat{{\varvec{p}}}. \end{aligned}$$

Note also that if ${\varvec{u}} \in \mathcal {M}_{\textbf{A}, {\varvec{c}}}$, then ${\varvec{x}}(t)=\bar{{\varvec{u}}}$ for all $t \ge 0$, i.e., it remains constant as the MLE.

We close the section with two remarks about possible variations of Construction 4.1.

### Remark 4.8

Networks that appear in biological systems are typically *bimolecular*, in the sense that the coefficients of each reactant complex sum up to at most 2. The maximum-likelihood-estimating networks of Construction 4.1 are not necessarily bimolecular, which could pose a challenge in their biochemical implementation. However, we note that they can always be modified into bimolecular networks after introducing additional auxiliary species and reactions, in such a way that the steady state concentrations in the original species still is the desired MLE (Edward 1981; Hernández-Bermejo and Fairén 1995; Wilhelm 2000; Plesa 2023).

### Remark 4.9

This section has been focused on proving that for a given log-affine model $\mathcal {M}_{\textbf{A},{\varvec{c}}}$, one can compute the MLE $\widehat{{\varvec{p}}}$ using the mass-action system $G_{\Lambda , {\varvec{c}}}$ in Construction 4.1. In some applications, it could be useful to also obtain the parameters ${\varvec{t}}=(t_1,\ldots ,t_d)$ that give rise to $\widehat{{\varvec{p}}}$ under the parametrization $\varphi _{\textbf{A},{\varvec{c}}}$ from (2.1). In order to do this, (Gopalkrishnan 2016) proposed an extension to $G_{\Lambda , {\varvec{c}}}$; the following is based on the same idea.

Choose a set $J\subseteq [m]$ of linearly independent columns of $\textbf{A}$ that span the column space. For each $i \in [d]$, add a new species $\textsf {Z}_i$; moreover for each $j \in J$, add the reactions

Notice that the added reactions do not affect any of the equations $\dot{x}_j$; hence the subsystem for $\dot{x}_j$ still converges to the MLE $\widehat{{\varvec{x}}} = \widehat{{\varvec{p}}}$. The added variables $z_i$ evolve according to $\dot{{\varvec{z}}}(t) = \sum _{j \in J} \left( x_j(t) - c_j {\varvec{z}}(t)^{{\varvec{a}}_j} \right) {\varvec{a}}_j$, which at steady state satisfies $\widehat{x}_j = c_j \widehat{{\varvec{z}}}^{{\varvec{a}}_j}$ for all $j \in J$. Hence, at steady state, we recover the parametrization $\widehat{{\varvec{x}}} = {\varvec{c}} \circ {\varvec{t}}^{\textbf{A}}$ for the subsystem on ${\varvec{x}}$. An analysis similar to that of Gopalkrishnan (Gopalkrishnan 2016, Theorem 6) shows that the augmented system is globally stable if and only if $G_{\Lambda ,{\varvec{c}}}$ is globally stable with respect to the appropriate stoichiometric compatibility class.

## Impact of the spanning set on the dynamics

A key step in Construction 4.1 is choosing a finite spanning set $\Lambda \subseteq \ker _{\mathbb {Z}}(\textbf{A})$ for $\ker (\textbf{A})$. In this section, we discuss how this choice influences the dynamical properties of the mass-action system $G_{\Lambda , {\varvec{c}}}$. We will focus on three properties: global stability (Sect. 5.1), deficiency (Sect. 5.2), and the rate of convergence (Sect. 5.3). The two main options we will consider are the following:

- If $\Lambda$ is chosen to be a *Markov basis*, we show in Sect. 5.1 that asymptotic stability can be strengthened to global stability, and in Sect. 5.3, we give examples where it ensures fast and uniform convergence.
- If $\Lambda$ is a *vector space basis* for $\ker (\textbf{A})$, the cardinality is minimal, which means the fewest possible reactions to be engineered in a synthetic biology context. In Sect. 5.2, we show that this implies that the deficiency is zero.

### Global stability and boundary steady states

An important question about our maximum-likelihood-estimating networks from the point of view of computational reliability is whether we have *global stability*, in the sense that the trajectory of the initial value problem in Theorem 4.6 converges to the MLE $\widehat{{\varvec{p}}}$ for any vector of counts ${\varvec{u}}\in \mathbb {R}_{\ge 0}^m$.

As pointed out in Sect. 3, the Global Attractor Conjecture states that we have global convergence for strictly positive vectors ${\varvec{u}}\in \mathbb {R}_{>0}^m$, but is only proven for special classes of networks, and does not directly address the case when ${\varvec{u}}\in \partial \mathbb {R}_{\ge 0}^m$.

In this section, we will focus on the property of *lacking relevant boundary steady states* (discussed at the end of Sect. 3) as a sufficient condition for global convergence. For $G_{\Lambda ,{\varvec{c}}}$, this is equivalent to the system

$$\begin{aligned} \sum _{{\varvec{\gamma }}\in \Lambda } \big ( {\varvec{c}}^{{\varvec{\gamma }}^+} {\varvec{x}}^{{\varvec{\gamma }}^-} - {\varvec{c}}^{{\varvec{\gamma }}^-} {\varvec{x}}^{{\varvec{\gamma }}^+}) \cdot {\varvec{\gamma }}={\varvec{0}}, \qquad \textbf{A}{\varvec{x}}=\textbf{A}{\varvec{u}} \end{aligned}$$

having no solution in $\partial \mathbb {R}_{\ge 0}^m$ for any ${\varvec{u}}\in \mathbb {R}_{>0}^m$.

#### Theorem 5.1

Let $\textbf{A}\in \mathbb {Z}^{d\times m}$ with $\mathbbm {1}\in {{\,\textrm{row}\,}}(\textbf{A})$, and ${\varvec{c}}\in \mathbb {R}_{>0}^m$, and suppose that $\Lambda \subseteq \ker _{\mathbb {Z}}(\textbf{A})$ is a finite spanning set of $\ker (\textbf{A})$ such that $G_{\Lambda ,{\varvec{c}}}$ lacks relevant boundary steady states. Then, for any vector of observed data ${\varvec{u}}\in \mathbb {R}_{\ge 0}^m$ such that $(\bar{{\varvec{u}}}+\ker (\textbf{A}))_{>0}$ is nonempty, the trajectory ${\varvec{x}}(t;\bar{{\varvec{u}}})$ converges to the MLE of ${\varvec{u}}$ in the model $\mathcal {M}_{\textbf{A},{\varvec{c}}}$.

#### Proof

This is a direct consequence of Proposition 3.13 and Theorem 4.6. $\square$

It is natural to ask whether the spanning set $\Lambda$ can be chosen in a way that rules out relevant boundary states, and thereby ensures global convergence. We prove that choosing $\Lambda$ to be a Markov basis achieves this.

#### Theorem 5.2

If $\Lambda \subseteq \ker _{\mathbb {Z}}(\textbf{A})$ is a Markov basis for $\textbf{A}$, then the mass-action system $G_{\Lambda , {\varvec{c}}}$ in Construction 4.1 lacks relevant boundary steady states. In particular, $G_{\Lambda , {\varvec{c}}}$ satisfies the global convergence property of Theorem 5.1.

#### Proof

By Proposition 3.12, the set of nonnegative steady states of $G_{\Lambda ,{\varvec{c}}}$ is given by

$$\begin{aligned} \mathcal {E}_{G_{\Lambda ,{\varvec{c}}}}^{\ge 0}=\Big \{{\varvec{x}}\in \mathbb {R}_{\ge 0}^m:{\varvec{c}}^{{\varvec{\gamma }}^-}{\varvec{x}}^{{\varvec{\gamma }}^+}={\varvec{c}}^{{\varvec{\gamma }}^+}{\varvec{x}}^{{\varvec{\gamma }}^-}\text { for all }{\varvec{\gamma }}\in \Lambda \Big \}=\overline{\mathcal {X}}_{\textbf{A},{\varvec{c}}}, \end{aligned}$$

where the second equality follows from Lemma 2.3. The lack of relevant boundary steady states now follows from the fact that, for any ${\varvec{u}}\in \mathbb {R}_{>0}^m$, the intersection $\overline{\mathcal {X}}_{\textbf{A},{\varvec{c}}}\cap ({\varvec{u}}+\ker (\textbf{A}))$ consists of a strictly positive point (and therefore has empty intersection with $\partial \mathbb {R}_{\ge 0}^m$) according to Theorem 2.5. $\square$

#### Remark 5.3

A similar statement appears in Gopalkrishnan (Gopalkrishnan 2016, Lemma 2) for ${\varvec{c}} = \mathbbm {1}$, with a different proof relying on siphon theory. The statement of Gopalkrishnan (2016) uses the weaker assumption that $\Lambda$ is a $\mathbb {Z}$-basis for the abelian group $\ker _{\mathbb {Z}}(\textbf{A})$, rather than a Markov basis, but as shown by Example 5.4(a) below, this is *not* sufficient to rule out relevant boundary steady states. Indeed, the key step in the proof given in Gopalkrishnan (2016) is to show that the so-called *associated ideal* $J {:}{=}\langle {\varvec{x}}^{\varvec{y}}-{\varvec{x}}^{{\varvec{y}}'}: {\varvec{y}}\rightarrow {\varvec{y}}'\in E \rangle$ of the network is prime, which proves structural persistence by Gopalkrishnan (Gopalkrishnan 2011, Theorems 4.1 and 5.2), and thereby rules out relevant boundary steady states. For $G_{\Lambda }$, we have $J=\langle {\varvec{x}}^{{\varvec{\gamma }}^+}-{\varvec{x}}^{{\varvec{\gamma }}^-}: {\varvec{\gamma }}\in \Lambda \rangle$. When $\Lambda$ is a Markov basis, primeness of *J* follows from the fact that toric varieties are irreducible, but when $\Lambda$ is not a Markov basis, *J* is always non-prime.

If $\Lambda \subseteq \ker (\textbf{A})$ is not a Markov basis, there might be additional solutions to the binomial equations ${\varvec{c}}^{{\varvec{\gamma }}^-}{\varvec{x}}^{{\varvec{\gamma }}^+}={\varvec{c}}^{{\varvec{\gamma }}^+}{\varvec{x}}^{{\varvec{\gamma }}^-}$ for ${\varvec{\gamma }}\in \Lambda$ compared to the binomial system induced by a Markov basis for $\textbf{A}$. However, it is only in some cases that these additional solutions constitute relevant boundary steady states. We illustrate this, as well as the concept of siphons and structural persistence, in the next example. Note that for a network $G_{\Lambda }$, a siphon corresponds to a set $Z\subseteq [m]$ such that for every ${\varvec{\gamma }}\in \Lambda$ and $i\in Z$ with $\gamma _i>0$, there is a $j\in Z$ such that $\gamma _j<0$. Structural persistence means that for every minimal siphon *Z*, there is some ${\varvec{a}}\in {{\,\textrm{row}\,}}(\textbf{A})\cap \mathbb {R}_{\ge 0}^m$ with ${{\,\textrm{supp}\,}}({\varvec{a}})=Z$.

#### Example 5.4

Consider again the log-linear model $\mathcal {M}_{\textbf{A}}$ from Examples 2.4 and 4.4, where

$$\begin{aligned} \textbf{A}=\begin{pmatrix}4& 2& 3& 1\\ 0& 2& 1& 3\end{pmatrix}. \end{aligned}$$

**Fig. 3:**
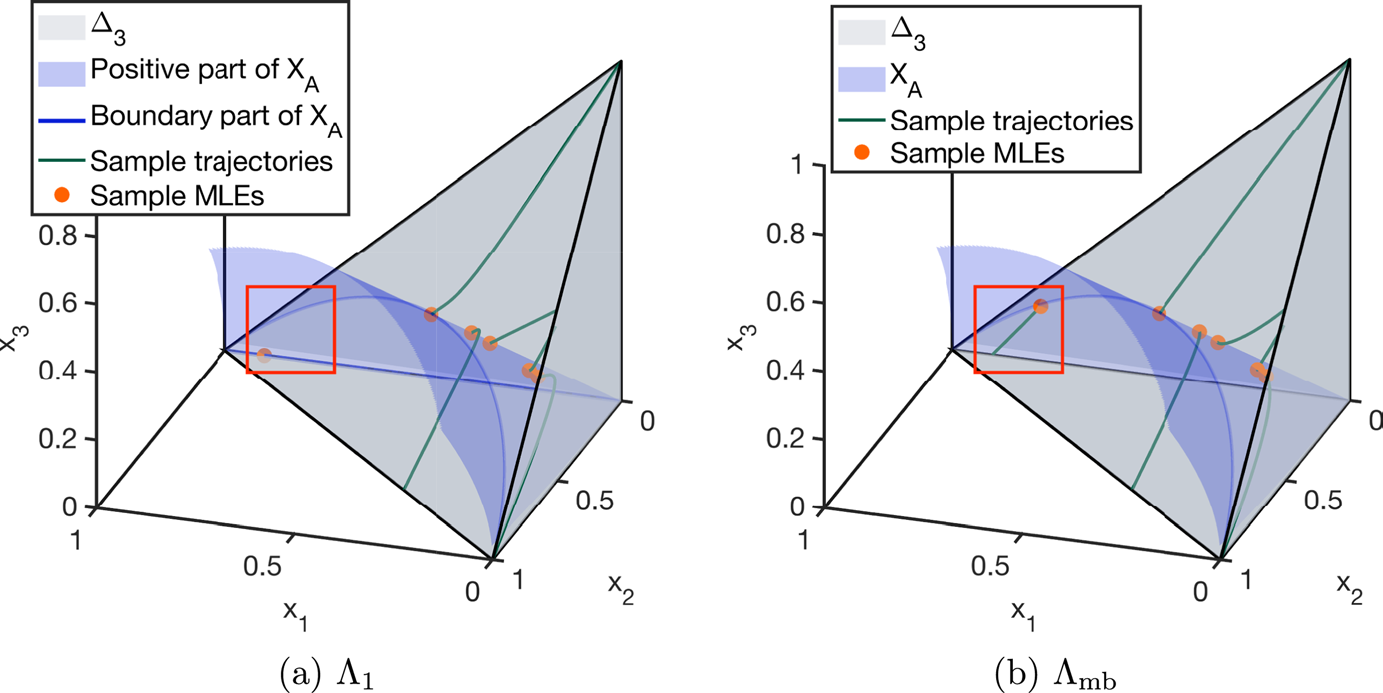
Illustration of Example 5.4, showing trajectories and steady states for several initial conditions under the canonical projection to $(x_1,x_2,x_3)$. Red boxes: The observed distribution $\bar{{\varvec{u}}} = (0.9,0,0,0.1)$ is a boundary steady state for system given by $\Lambda _1$, while the system given by $\Lambda _\textrm{mb}$ evolves from $\bar{{\varvec{u}}}$ to the MLE

- Recall the non-Markov basis $\Lambda _1 =\{(1,1,-2,0), (0,2,-1,-1)\}$, which is a $\mathbb {Z}$-basis for $\ker _{\mathbb {Z}}(\textbf{A})$. From this, we obtain the mass-action system in (3.2), where all the rate constants are 1. This system has relevant boundary steady states, e.g., $\widehat{{\varvec{x}}} = (0.9,0,0,0.1)$; see red box in Fig. 3a. In this case, $\{2,3\}$ is a minimal siphon, but there are no vectors ${\varvec{w}}\in {{\,\textrm{row}\,}}(\textbf{A})$ with ${{\,\textrm{supp}\,}}({\varvec{w}})=\{2,3\}$, so the siphon criterion is inconclusive.
- For the Markov basis $\Lambda _\textrm{mb} =\{(1,1,-2,0)$, $(0,2,-1,-1)$, $(2,0,-3,1)$, $(1,-1,-1,1)\}$, we obtained the mass-action system (4.1) with four pairs of reversible reactions. By Theorem 5.2, $G_{\Lambda }$ lacks relevant boundary steady states, and we are guaranteed to have global convergence. This is illustrated in Fig. 3b.
- Finally, for the non-Markov basis $\Lambda _4=\{ (1,-3,0,2), (-1,-1,2,0)\}$, which is also a $\mathbb {Z}$-basis for $\ker _{\mathbb {Z}}(\textbf{A})$, we obtain a structurally persistent network; the minimal siphons are $\{1,2,3\}$ and $\{2,3,4\}$, which are the supports of the vectors $(12,4,8,0),(0,2,1,3)\in {{\,\textrm{row}\,}}(\textbf{A})$. Hence, it follows from Propositions 3.9 and 3.11 that we have global convergence.

### Deficiency

A challenge in the chemical implementation of $G_{\Lambda ,{\varvec{c}}}$ is fine-tuning the rate constants to the values prescribed by Construction 4.1. Because of this, an important question is to what extent the dynamical properties of $G_{\Lambda ,{\varvec{c}}}$ are preserved under perturbations to the rate constants.

In this section, we will focus on the particularly well-behaved scenario of deficiency zero. The deficiency of a (weakly) reversible reaction network $G=(V,E)$ is $\delta := |V| - \ell - \dim (S)$, where $\ell$ is the number of connected components and *S* is the stoichiometric subspace. By Craciun et al. (2009), $\delta$ is the codimension of the subset of the rate constants for which *G* is complex-balanced (and hence satisfies analogous properties as those given in Theorem 3.5 and v 3.13). In particular, if $\delta =0$, the Deficiency Zero Theorem (Horn 1972) states that the network is complex-balanced for *all* values of the rate constants. For a more in-depth introduction to deficiency theory, we refer to Feinberg (2019).

#### Theorem 5.5

Let $\textbf{A}\in \mathbb {Z}^{d\times m}$ and let $\Lambda \subseteq \ker _{\mathbb {Z}}(\textbf{A})$ be a finite spanning set of $\ker (\textbf{A})$.

- The deficiency of $G_{\Lambda }$ is $\delta =|\Lambda |-r-\dim (\ker (\textbf{A}))$, where $|\Lambda |$ is the cardinality of $\Lambda$ and *r* is the cycle rank (the number of independent cycles) in the underlying undirected graph of $G_{\Lambda }$.
- If $\Lambda$ is a vector space basis for $\ker (\textbf{A})$, then the undirected graph of $G_{\Lambda }$ is acyclic and $\delta =0$.

#### Proof

Part (a) follows from the general graph-theoretic fact that the number of edges minus the cycle rank equals the number of vertices minus the number of connected components. Part (b) follows from part (a), combined with the fact that $\delta$ cannot be negative. $\square$

Note that $G_{\Lambda }$ has deficiency zero precisely when the cycle rank equals the number of vectors in $|\Lambda |$ that are redundant for spanning $\ker (\textbf{A})$. When this property of having deficiency zero coincides with the property of lacking relevant boundary steady states discussed in Sect. 5.1 (e.g., when $\Lambda$ is both a vector space basis and Markov basis), then the network displays very robust dynamics.

#### Corollary 5.6

Suppose that $\Lambda \subseteq \ker _{\mathbb {Z}}(\textbf{A})$ is such that $\delta =0$ and $G_{\Lambda }$ lacks relevant boundary steady states for all rate constants. Then $G_{\Lambda }$ has a unique and globally stable positive steady state in each stoichiometric compatibility class for every choice of rate constants.

#### Proof

This follows by the complex-balanced analog of Proposition 3.13, combined with the Deficiency Zero Theorem (Horn 1972, Theorem 4A). $\square$

#### Example 5.7

Consider again the choices of spanning sets discussed in Example 5.4. Using $\Lambda _1$ gives a network with $\delta =0$, $\Lambda _{\operatorname {mb}}$ gives a network with $\delta =1$, and $\Lambda _4$ gives a network with $\delta =0$ that satisfies the property in Corollary 5.6.

### Rate of convergence

In the neighborhood of a hyperbolic equilibrium, the rate of convergence is dictated by the eigenvalue $\lambda _{\max }$ with the least negative real part for the Jacobian matrix (Chicone 2024), assuming all its eigenvalues have negative real parts. Convergence along the eigendirection for $\lambda _{\max }$ is the slowest; thus the more negative $\textrm{Re}(\lambda _{\max })$ is, the faster the system converges to equilibrium, with a rate of convergence $-\textrm{Re}(\lambda _{\max })$. For detailed-balanced systems, we only consider its nonzero eigenvalues, since the detailed-balanced steady state is hyperbolic with respect to its stoichiometric compatibility class (Siegal and Johnston 2015).

In this section, we explore how the rate of convergence depends on the choice of the spanning set, using as two examples: the binary 4-cycle (Example 2.8), and the independence model with design matrix $\textbf{A}_\textrm{ind}(10,10)$ (Example 2.7). (Similar observations were made for $\textbf{A}_\textrm{ind}(r_1,r_2)$ with $3 \le r_1 \le 10$ and $2 \le r_2 \le 10$, and for scaling factors other than $\mathbbm {1}$.) Each of these models has the property that there is a unique Markov basis $\Lambda _\textrm{mb}\subseteq \ker _{\mathbb {Z}}(\textbf{A})$ of minimal cardinality; see the database Markov-Bases.de [4t] and (Drton et al. 2008, Proposition 1.2.2).

In our experiments, we first constructed a strictly increasing sequence of subsets

$$\begin{aligned} \Lambda _1\subsetneq \Lambda _2 \subsetneq \cdots \subsetneq \Lambda _9\subsetneq \Lambda _{10} \end{aligned}$$

where $\Lambda _1$ is a vector space basis for $\ker (\textbf{A})$, and $\Lambda _{10}$ has the same cardinality as $\Lambda _\textrm{mb}$. Then for each $\Lambda _i$ and for a random choice of initial conditions ${\varvec{u}} \in \Delta _{m-1}$, we approximated the positive steady state, i.e., the MLE, and the nonzero eigenvalues of the Jacobian matrix. Thus we estimated the rate of convergence $-\textrm{Re}(\lambda _{\max })$ of the system $G_{\Lambda _i, \mathbbm {1}}$. We conducted two numerical experiments: (i) where $\Lambda _{10}$ is the unique Markov basis of minimal cardinality, and (ii) where $\Lambda _{10}$ is a random spanning set^1^ . Detailed numerical results and codes for the numerical experiments are available at https://github.com/oskarhenriksson/estimating-birch-points-with-networks.^2^

**Fig. 4:**
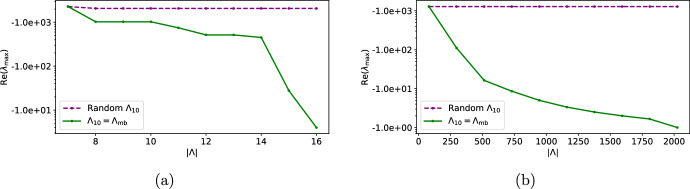
The least negative nonzero eigenvalues for systems constructed using increasing sequences of spanning sets $\Lambda _i$, which increase towards either (solid) the unique Markov basis $\Lambda _{10} = \Lambda _{\textrm{mb}}$, or (dashed) a random spanning set. The models considered were (**a**) the binary 4-cycle model (Example 2.8), and (**b**) the independence model (Example 2.7) with $r_1 = 10$ and $r_2 = 10$

Figure 4 is a plot of the real parts of the least negative nonzero eigenvalues $\textrm{Re}(\lambda _{\max })$ for each $\Lambda _i$, where the results for the binary 4-cycles are shown in (a), and those for the independence model are shown in (b). Notice that for each model, for a fixed number of reactions, i.e., for fixed $|\Lambda _i|$, the rate of convergence is faster when $\Lambda _i$ is increasing towards a Markov basis, compared to when they came from a random spanning set. The numerical experiments suggest that one cannot speed up the rate of convergence by simply adding more vectors to the spanning set, unless the vectors are chosen wisely, e.g., building up towards a Markov basis. Understanding this interplay between algebraic geometry and dynamics is an interesting direction for future research.

**Fig. 5:**
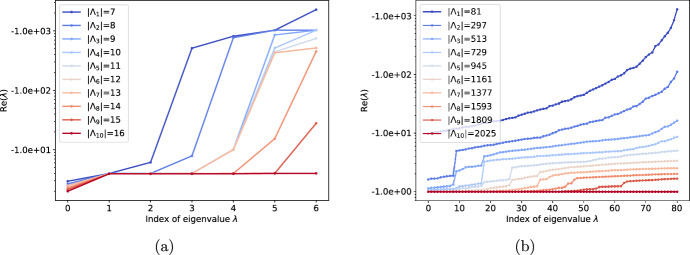
All nonzero eigenvalues of the systems constructed using $\Lambda _i$, which increases towards the Markov basis $\Lambda _{10}$ for (**a**) the binary 4-cycle model (Example 2.8), and (**b**) the independence model (Example 2.7) with $r_1 = 10$ and $r_2 = 10$. As $\Lambda _i$ increases towards the Markov basis, all nonzero eigenvalues tend to become more negative

When the spanning set $\Lambda _i$ increases towards the Markov basis, we observed in these two examples that not only is the least negative nonzero eigenvalue monotonically decreasing (i.e., increasing rate of convergence), but that *all* eigenvalues tend to be more negative. Figure 5 shows all the nonzero eigenvalues for (a) the binary 4-cycle model and (b) independence model. The phenomenon is more pronounced in the case of the independence model (Fig. 5b), where we observed that all the eigenvalues are $-1$ for the minimal Markov basis, i.e., the system converges to the MLE globally with rate of convergence 1.

We conclude this section by proving that the numerical observation concerning the independence model holds more generally: the detailed-balanced system constructed using the unique minimal Markov basis always has global rate of convergence 1. In fact, we give explicit analytical solutions for the independence model, which shows that the rate of convergence is *globally* exponential with rate 1, not just near the MLE. This is a highly desirable feature from a molecular computation point of view, since it means that the system will converge to the MLE with a rate that is independent of the input (see, e.g., the discussion in Anderson and Joshi (2024)). See also Example 4.7 for another example of when a Markov basis gives rise to a global rate of convergence. An interesting problem for future work is to investigate whether we can always find closed form formulas for models where the MLE depends rationally on the observed distribution.

#### Proposition 5.8

Consider the initial value problem constructed by Construction 4.1 using the unique minimal Markov basis (2.6) for the independence model with $r_1$ and $r_2$ states:

5.1 $$\begin{aligned} \dot{p}_{ij}=\sum _{k\in [r_1]\setminus \{i\}}\sum _{\ell \in [r_2]\setminus \{j\}} (p_{i\ell }p_{kj}-p_{ij}p_{k\ell }),\quad p_{ij}(0)=\bar{u}_{ij},\quad (i,j)\in [r_1]\times [r_2]. \end{aligned}$$

The unique solution to (5.1) is given explicitly by

5.2 $$\begin{aligned} p_{ij}(t)=\frac{1}{u_{++}^2}\Big (u_{i+}u_{+j}+(u_{ij}u_{++}-u_{i+}u_{+j})e^{-t}\Big ), \end{aligned}$$

where

$$\begin{aligned} u_{+j}{:}{=}\sum _{i=1}^{r_1} u_{ij},\quad u_{i+}{:}{=}\sum _{j=1}^{r_2} u_{ij},\quad \text {and}\quad u_{++}{:}{=}\sum _{i=1}^{r_1}\sum _{j=1}^{r_2} u_{ij}. \end{aligned}$$

#### Proof

Observe that

$$\begin{aligned} \lim _{t\rightarrow \infty } p_{ij}(t)=\frac{u_{i+}u_{+j}}{u_{++}^2}, \end{aligned}$$

which is consistent with the known MLE (2.7) for independence models. Furthermore, we have $p_{ij}(0)=\frac{u_{ij}}{u_{++}} = \bar{u}_{ij}$, so it suffices to show that substituting (5.2) into the right-hand side of the ODEs (5.1), recovers the derivatives $\dot{p}_{ij}$, which are given by

$$\begin{aligned} \dot{p}_{ij}=\frac{(u_{i+}u_{+j}-u_{ij}u_{++})}{u_{++}^2}e^{-t}. \end{aligned}$$

The proof proceeds by direct computation, involving the identities

$$\begin{aligned} \sum _{k\in [r_1]\setminus \{i\}}\sum _{\ell \in [r_2]\setminus \{j\}} u_{kl} = u_{++}-u_{i+}-u_{+j}+u_{ij} \end{aligned}$$

and

$$\begin{aligned} u_{i+}u_{+j}-u_{ij}u_{++}=\sum _{k\in [r_1]\setminus \{i\}}\sum _{\ell \in [r_2]\setminus \{j\}} (u_{i\ell }u_{kj}-u_{ij}u_{k\ell }). \end{aligned}$$

$$\square$$

## Conclusion and outlook

In this work, we describe a method that, for a given statistical log-affine model $\mathcal {M}_{\textbf{A},{\varvec{c}}}$, returns a mass-action system $G_{\Lambda ,{\varvec{c}}}$ with the property that the MLE $\widehat{{\varvec{p}}}$ of $\mathcal {M}_{\textbf{A},{\varvec{c}}}$ with respect to an observed distribution $\bar{{\varvec{u}}}$ is the unique positive steady state of $G_{\Lambda ,{\varvec{c}}}$ in the stoichiometric compatibility class of $\bar{{\varvec{u}}}$.

Throughout the paper, we see examples of how $G_{\Lambda ,{\varvec{c}}}$ is dynamically more well-behaved if $\Lambda$ is chosen as a Markov basis for the design matrix $\textbf{A}$. In particular, for a Markov basis, there are no relevant boundary steady states, which guarantees global convergence to the MLE. Taking $\Lambda$ to be a $\mathbb {Z}$-basis is not enough to rule out the existence of relevant boundary steady states, as shown in Example 5.4. Furthermore, the numerical experiments presented in Sect. 5.3 suggest that convergence to the MLE is faster when using a Markov basis than a vector space basis. For example, for independence models, the minimal Markov bases always give global rates of convergence 1, while with a vector space basis, the system has components whose time-scales are as high as $10^3$, which could lead to unreliable computational performance.

From a synthetic biology point of view, these observations suggest an interesting trade-off between desirable dynamics and feasibility of chemically implementing a given network, since a Markov basis is typically much larger than a vector space basis for $\ker (\textbf{A})$. For instance, in the independence model case, the smallest Markov basis for $\textbf{A}_\textrm{ind}(r_1,r_2)$ has $$\left( {\begin{array}{c}r_1\\ 2\end{array}}\right) \left( {\begin{array}{c}r_2\\ 2\end{array}}\right)$$ elements, while the dimension of the kernel is $(r_1-1)(r_2-1)$. An interesting direction for future research is therefore to find systematic ways of choosing spanning sets $\Lambda$ that are not Markov bases, but still give rise to well-behaved dynamics, similarly to what was achieved in Example 5.4(c).

## Data Availability

The code and simulated data used in the computational experiments in Sect. 5.3 is available in the repository https://github.com/oskarhenriksson/estimating-birch-points-with-networks.

## Footnotes

1 Obtained by extending $\Lambda _1$ with linear combinations of vectors in $\Lambda _1$ whose coefficients were uniformly drawn from $\{-1,0,1\}$.

2 The code is based on Python 3.10.12, with packages NumPy 1.26.4 (Harris et al. 2020), Scipy 1.11.4 (Virtanen et al. 2007), and the reaction networks theory package Tellurium 2.2.10 (Kiri Choi 2018).
